# The interaction of MD-2 with small molecules in huanglian jiedu decoction play a critical role in the treatment of sepsis

**DOI:** 10.3389/fphar.2022.947095

**Published:** 2022-09-09

**Authors:** Guirong Chen, Xiaobo Wang, Chang Liu, Mingbo Zhang, Xueying Han, Yubin Xu

**Affiliations:** ^1^ College of Pharmacy, Liaoning University of Traditional Chinese Medicine, Shenyang, China; ^2^ Taizhou Central Hospital (Taizhou University Hospital), Taizhou, China; ^3^ Institute of Pharmacy, 967th Hospital of the Joint Logistics Support Force of the Chinese People’s Liberation Army, Dalian, China

**Keywords:** Huanglian Jiedu Decoction, active components, anti-sepsis, myeloid differentiation protein-2, LPS-TLR4/MD-2-NF-κB

## Abstract

Huanglian Jiedu Decoction (HJD) is used for treating sepsis in China. Active components from HJD refer to various active ingredients of HJD, while active component formulation (ACF) refers to the combination of palmatine, berberine, baicalin, and geniposide from HJD according to the quantity of HJD. The detailed mechanisms of the active components from HJD and ACF in sepsis treatment are unclear. Molecular docking, surface plasmon resonance (SPR), ELISA, RT-qPCR, and Western blotting were used to assay the possible mechanism *in vitro*. The efficacy and mechanism of ACF and HJD were assessed by pharmacodynamics and metabolomics analyses, respectively. The results revealed that palmatine, berberine, baicalin, and geniposide showed good binding capacity to MD-2; decreased the release of NO, TNF-α, IL-6, and IL-1β; inhibited the mRNA expression of iNOS, TNF-α, IL-6, IL-1β, and COX-2; and downregulated the protein expressions of MD-2, MyD88, p-p65, and iNOS induced by LPS; which indicated that they can inactivate the LPS-TLR4/MD-2-NF-κB pathway. Thus, ACF was formed, and the pharmacodynamics assay suggested that ACF can reduce inflammatory cell infiltration and organ damage in accordance with HJD. Furthermore, 39 metabolites were selected and identified and the regulatory effect of these metabolites by ACF and HJD was almost consistent, but ACF might alleviate physical damage caused by HJD through regulating metabolites, such as 3-hydroxyanthranilic acid. ACF could represent HJD as a new formulation to treat sepsis.

## 1 Introduction

Sepsis is a class of systemic inflammatory response syndrome caused by the failure of the host response to infection, which has currently become one of the main causes of death in critical patients (Kaukonen et al., 2015; Singer et al., 2016; Arefian et al., 2017). Globally, there are more than 18 million sepsis patients annually, approximately 30–40% of which would progress to severe sepsis or septic shock, with a hospital mortality of 25–30% and 40–70%, respectively (Nolan and Weiden, 2015). Recently, the understanding of sepsis pathogenesis has become sharper, but the effective treatment for sepsis is still far from reaching prospective outcomes, with no clinically effective drug for treating sepsis at present. Therefore, developing effective drugs and methods to treat sepsis is of high clinical importance.

Numerous studies have verified that lipopolysaccharide (LPS), the major component of the outer membrane of Gram-negative bacteria, is a key pattern-recognition molecule for sepsis (Victor et al., 2004; Lin and Yeh, 2005). LPS involves an O-specific polysaccharide chain, a core polysaccharide, and a lipid A, comprising the biological active sites of endotoxins (Takeda, 2010). After transporting by lipopolysaccharide-binding protein (LBP), LPS binds to CD14, a glycoprotein anchored at mononuclear/macrophage cell membranes, generating an intracellular cascade that determines cellular fate (Cighetti et al., 2014), and signal transduction should be along with Toll-like receptor 4 (TLR4) and myeloid differentiation factor 2 (MD-2) (Wang et al., 2012; Rajaiah et al., 2015). MD-2 is a key molecule of TLR4 for recognition of LPS signaling, and its expression of functional domains could bind with TLR4 to form TLR4/MD-2 complexes, which played a key regulatory role in TLR4 activation. The other part was released into the plasma to form soluble MD-2, which bound with LPS through CD14 participation (Chi et al., 2013; Vilades et al., 2014; Hosoki et al., 2016). The structural integrity of receptor complexes which usually comprise MD-2, TLR4, and CD14 were the bases for recognition and subsequent signal transduction of the LPS signal pathway. When there is disruption of any functional domain, signal transduction would be disordered. Some studies had shown that blocking LPS binding to MD-2 is nearly 100 times easier than blocking LPS binding to TLR4/MD-2, also indicating that MD-2 and the domain of MD-2 bound with LPS play a decisive role in the whole signal transduction (Akashi et al., 2003). Therefore, the LPS-TLR4/MD-2-NF-κB pathway is a critic signaling pathway for sepsis (Park et al., 2012). It might be a more effective therapeutic strategy to develop a drug that can interfere with or inhibit LPS binding to MD-2 for controlling the inflammatory response induced by LPS.

TCM indicates that there are two major syndromes of noxious heat type and Stasis-toxin Internal Obstruction Pattern in the early stage of sepsis (Wang et al., 2016). HJD is mainly used to treat all kinds of noxious heat type for its good clearing heat and purging fire capacity and removing toxins of traditional efficacy (Fang, 2015). HJD, the classical formulation for relieving fever and toxicity, is composed of four herbs: *Coptis chinensis* Franch., *Scutellaria baicalensis* Georgi, *Phellodendron amurense* Rupr., and *Gardenia jasminoides* J. Ellis and has been used for treating sepsis in China for many years (Chen G. et al., 2016). Clinical and animal assays indicated that HJD shows strong activity in the sepsis model, including sepsis patients (Wang et al., 2017) and rats induced with cecal ligation and puncture (CLP) (Lv et al., 2017). Four active components: palmatine, berberine, baicalin, and geniposide from HJD were screened based on lipid A target by biosensor technology and showed high neutralized activity of LPS. *In vitro* and *in vivo* studies found that they play an anti-sepsis role through regulating TLR4-NF-κB by binding to lipid A, which is the active center of LPS (Chen GR. et al., 2016). However, the signaling pathway of the effective components on anti-sepsis needs further exploration. In this work, MD-2, the key protein of the TLR4-NF-κB pathway, was used as a potential target for further study based on the LPS-TLR4/MD-2-NF-κB signaling pathway. The binding of the four active components to MD-2 was assayed by molecular docking and SPR techniques, which was also verified by *in vitro* activity studies. Then, the four active components were prepared as ACF according to the original content of HJD prescription. The efficacy and mechanism of ACF and HJD were evaluated through pharmacodynamics and metabolomics analysis, respectively. The study will clarify the mechanism of the effective components on sepsis and lay a foundation for further development of anti-sepsis agents.

## 2 Materials and methods

### 2.1 Materials

LPS: MD-2; palmatine, berberine, baicalin, and geniposide were isolated from HJD by the authors in the previous study, and their purity were determined to be >98% by HPLC (Chen G. et al., 2016). All other reagents and chemicals used in the study were of analytical grade.

### 2.2 *In vitro* evaluation of palmatine, berberine, baicalin, and geniposide from HJD

#### 2.2.1 The evaluation of palmatine, berberine, baicalin, and geniposide bound to MD-2 by molecular docking

AutoDock Vina (Scripps Research Institute, San Diego, CA, United States ) was used to evaluate the binding of baicalin, palmatine, berberine, and geniposide with MD-2. The X-ray crystal structure of human MD-2 complexed with lipid IVa (PDB 3vq2, 2.48 Å) was obtained from the Protein Data Bank. The protonation state of the protein and the ligand were prepared using default settings. A grid box with dimensions of 60 x ×60 x ×52 Å (-12.13, 22.813, -2.759) with a spacing of 0.375 Å was constructed around the docking area using Autogrid 4.2 software. Molecules were docked using Vina with exhaustiveness grade 8. The lowest energy conformations were selected, and the ligand interactions with MD-2 were determined and visualized with Accelrys Discovery Studio Visualizer 4.0 (Accelrys, San Diego, CA, United States ) and Pymol 0.99, respectively.

#### 2.2.2 The evaluation of palmatine, berberine, baicalin, and geniposide bound to MD-2 by SPR technology

Measurements were performed on an Octet RED96E by FortéBIO. Data acquisition was set to 5 Hz. The eight channels were equipped with Super Streptavidin (SSA) biosensors obtained from FortéBIO. MD-2 was biotinylated by EZ-Link NHS-PEG4-Biotin (Thermo Scientific) following the manufacturer’s guidelines. A 3:1 molar coupling ratio (reagent/protein) was used during the coupling, and the reaction was carried out in a standard assay buffer. The binding assay was performed in standard 96-well plates (black, polypropylene, 392 µl/well) by Greiner using sample volumes of 200 µl. Baicalin, palmatine, berberine, and geniposide were diluted into solutions in a buffer containing 10 mM PBS pH 7.5, 0.5% Tween20, and 0.5% DMSO, at concentrations of 1,000, 500, 250, 125, 62.5, 31.3, and 15.6 μM. A baseline step of 60 s, an association step of 45 s, and a dissociation step of 60 s were acquired using the protein-loaded and the control sensors for each point of the concentration series. Prior to analysis, the sensors were pre-wet in buffer. Then, six sensors were loaded with biotinylated MD-2 at a concentration of 10 μg/ml. Two of the sensors were blocked with 10 μg/ml biocytin and used as reference sensors to check for non-specific binding. Equilibration was performed in standard buffer. Association- and dissociation-sensorgrams were recorded, and by applying a global 1:1 fit to the raw data, the following KD-values could be determined: compounds: KD = 1.7 mM, Chi^2^ = 0.16, and *R* ^2^ = 0.98. Data were processed to remove the drift and well-to-well artifacts by using the ForteBio Data Analysis Software 10.0 (Molecular Devices, LLC, San Jose, CA, United States ).

#### 2.2.3 The pathway analysis of palmatine, berberine, baicalin, and geniposide in sepsis

The RAW 264.7 cell line was obtained from Procell life Science &Technology (Wuhan, China). Cells were grown as a monolayer in Dulbecco’s modified Eagle’s medium (DMEM; San Angelo, TX) supplemented with 10% fetal bovine serum (FBS; San Angelo, TX), penicillin (100 U/ml), and streptomycin (100 μg/ml) and maintained at 37°C in humidified atmosphere containing 5% CO_2_.

RAW 264.7 cells were seeded in a 24-well plate for 24 h at a density of 1.0 × 10^5^ cells/well. The cells were then incubated with fresh medium containing various palmatine, berberine, baicalin, or geniposide concentrations (8, 40, and 100 μg/ml) for 2 h. After the indicated incubation time, the cells were treated with LPS (100 ng/ml) for additional 24 h, and the supernatant was collected and analyzed for the presence of TNF-α, IL-6, NO, and IL-1β by ELISA according to the manufacturer’s protocol (Jian Cheng Bioengineering Institute, Nanjing, China).

Cells treated with 100 μg/ml palmatine, berberine, baicalin, or geniposide were collected for RT-q PCR and Western blotting experiments. The total RNA of cells was extracted using TRIzol reagent. For cDNA synthesis, an AMV First-Strand cDNA Synthesis Kit was used. The primer sequences of the primers for β-actin, IL-1β, IL-6, cyclooxygenase-2 (COX-2), inducible NO synthase (iNOS), and TNF-α are presented in Table 1. RT-q PCR analysis (all reagents were purchased from Tiangen Biotech Co., Ltd., Beijing, China) was performed as previously described (Chen et al., 2017). Briefly, the reaction conditions were 50°C for 2 min, 95°C for 10 min, 95°C for 30 s, and 60°C for 30 s for 40 cycles. The relative expression was quantified using the 2-ΔΔct analysis method with β-actin as the endogenous control.

**TABLE 1:** Primer sequences for RT-q PCR.

Name	Primer	Sequence	Size
β-actin	Forward	5‘- CAC​GAT​GGA​GGG​GCC​GGA​CTC​ATC -3’	240 bp
	Reverse	5‘- TAA​AGA​CCT​CTA​TGC​CAA​CAC​AGT -3’	
Mus IL1β	Forward	5‘- TCA​GGC​AGG​CAG​TAT​CAC​TC -3’	250 bp
	Reverse	5‘- AGC​TCA​TAT​GGG​TCC​GAC​AG -3’	
Mus IL6	Forward	5‘- GTT​GCC​TTC​TTG​GGA​CTG​AT -3’	131 bp
	Reverse	5‘- ATT​AAG​CCT​CCG​ACT​TGT​GA -3’	
Mus COX-2	Forward	5‘- ATC​ATA​AGC​GAG​GAC​CTG​GG -3’	205 bp
	Reverse	5‘- TCA​GGG​ATG​TGA​GGA​GGG​TA -3’	
Mus iNOS	Forward	5‘- TTG​GCT​CCA​GCA​TGT​ACC​CT -3’	121 bp
	Reverse	5‘- TCC​TGC​CCA​CTG​AGT​TCG​TC -3’	
Mus TNF-a	Forward	5‘- CGT​CAG​CCG​ATT​TGC​TAT​CT -3’	206 bp
	Reverse	5‘- CGG​ACT​CCG​CAA​AGT​CTA​AG -3’	

As to Western blotting assay, the cells were lysed using the RIPA lysis buffer (Beyotime Institute of Biotechnology, Haimen, China), and the nucleus and cytosol proteins were isolated using a nuclear protein isolation–translocation assay kit (Beyotime Institute of Biotechnology, Haimen, China). Then, the protein concentration of each well was determined by a BCA protein assay kit (Wuhan Sanying Biotechnology Co.,, Ltd., Wuhan, China). The protein extracts were separated within 10% SDS-polyacrylamide gel electrophoresis, transferred to PVDF membranes, blocked within five blocking solutions at room temperature for 2 h, and followed by incubation with primary antibodies (all purchased from Bioss Antibodies, Inc., Woburn, MA, United States ) overnight at 4°C. The membranes were rinsed with TBST six times, incubated with the HRP-conjugated secondary antibodies (Wuhan Boster Biological Technology Co.,, Ltd., Wuhan, China) at 37°C for 2 h, and visualized by an ECL (Pierce Chemical, Dallas, TX, United States ) method. The intensity of each protein band was measured by Image-Pro Plus 6.0 software (Media Cybernetics, Inc., Rockville, MD, United States ).

### 2.3 Pharmacodynamics and metabolomics analysis of ACF and HJD

#### 2.3.1 Preparation of ACF and HJD

The crude decoction pieces of *Coptis chinensis* Franch., *Scutellaria baicalensis* Georgi, *Phellodendron amurense* Rupr., and *Gardenia jasminoides* J. Ellis were purchased from Liaoning China Pharmacy Co.,, Ltd. and identified by professor Yanjun Zhai (College of pharmacy, Liaoning University of Traditional Chinese Medicine). The abovementioned four herbs were ground to powder or pieces and mixed in the ratio of 3:2:2:3 (weight: 540, 360, 360, and 540 g), and the mixture was then extracted by hot reflux extraction using water as the extraction solvent. The water extract was filtered, and the residue was further extracted again. All filtrates were combined and concentrated in a rotary evaporator to obtain 440.28 g dried powder of HJD. The yield of HJD was 24.46%. The contents of palmatine, berberine, baicalin, and geniposide in the HJD were 0.475, 0.985, 1.620, and 2.040%, respectively. 200 g dry powder of HJD was used for silica gel column chromatography for isolation of active compounds by gradient elution with CH_2_Cl_2_-MeOH. Palmatine, berberine, baicalin, and geniposide were isolated from HJD by silica gel column chromatography, and their purities were determined to be >98% by HPLC (Chen GR. et al., 2016). The yields of palmatine, berberine, baicalin, and geniposide in the HJD were 9.778, 13.079, 15.470, and 14.533%, respectively. HPLC fingerprinting was constructed as a means of quality control for HJD (Supplementary Figure S1). The ACF was composed by 3.5 mg of palmatine, 7.2 mg of berberine, 11.8 mg of baicalin, and 14.8 mg of geniposide according to the content of each compound in HJD, which was the same as the dosage of 726 mg HJD powder.

#### 2.3.2 Animal experiments and sample collection

The animal experiment was conducted in compliance with the ARRIVE guidelines and carried out in accordance with the U.K. Animals (Scientific Procedures) Act, 1986 and associated guidelines, EU Directive 2010/63/EU for animal experiments. Specific pathogen-free female Sprague–Dawley (SD) rats (220±20 g) were purchased from Liaoning Changsheng Biotechnology Co.,, Ltd., laboratory animal license SCXK (Liao): 2015-0001. The animals were housed in the Center for Animal Experiments of Liaoning University of Traditional Chinese Medicine (Shenyang, China). All rats received a standard laboratory diet for a 1-week acclimatization period. The experimental procedures were approved by Liaoning Provincial Animal Welfare in accordance with the National Institutes of Health guide for the care and use of laboratory animals (NIH Publications No. 8023, revised 1978).

Rats were randomly allocated into four groups, namely, control group, LPS group, HJD group (330 mg/kg), and ACF group (equivalent to 330 mg/kg of HJD) with eight rats in each group (Zang et al., 2018). The HJD group was administrated with HJD 330 mg/kg/d, and 660 mg dry extract of HJD was dissolved in 20 ml of 0.5% CMC-Na solution to obtain HJD decoction of 330 mg/ml. All the rats were injected intraperitoneally with a dosage of 22 mg/kg LPS to establish sepsis models, while the rats from the control group were administrated with normal saline. 6 h after the model was established, rats in the HJD and ACF groups were given intragastric administration of the corresponding test substances, and rats in the control and LPS groups received 0.5% CMC-Na solution. 24 h after modeling, the rats were anesthetized by pentobarbital, and blood samples were obtained and then centrifuged at 3,000 rpm for 10 min to obtain serum for ELISA assay. The rats were killed after blood collection, and then the heart, lung, liver, kidney, and colon tissues were collected and stored at -80°C for further assays.

#### 2.3.3 Histopathological studies

Histopathological studies were performed according to the previous literature (Chen et al., 2020). Briefly, the samples of the heart, lung, liver, kidney, and colon tissues were fixed in 10% phosphate-buffered formalin. Then, these tissues were dehydrated, embedded, sliced, and stained with hematoxylin and eosin (H & E). The slides were visualized using a light microscope (Nikon, Japan).

#### 2.3.4 ELISA assay for NO, IL-1β, IL-6, and TNF-α levels in serum

The NO, IL-1β, IL-6, and TNF-α levels in serum of the control group, LPS group, HJD group, and ACF group were determined by ELISA kits following the manufacturer’s instructions, respectively.

#### 2.3.5 Metabolomics

Metabolomics experiments were performed as in previous studies (Dunn et al., 2011). Briefly, a 100-μl serum sample from rats in each group was transferred to an EP tube, and 400 μl extract solution (acetonitrile: methanol = 1:1) containing internal standard (L-2-chlorophenylalanine (Shanghai Hengbai Biotechnology Co.,, Ltd.), 2 μg/ml) was added. After 30-s vortexing, the samples were sonicated for 10 min in ice-water bath. Then, the samples were incubated for 1 h at -40°C and centrifuged at 10,000 rpm for 15 min at 4°C. 400 μl of the supernatant was transferred to a fresh tube and dried in a vacuum concentrator at 37°C. Then, the dried samples were reconstituted in 200 μl of 50% acetonitrile by sonication on ice for 10 min. The constitution was then centrifuged at 13,000 rpm for 15 min at 4°C, and 75 μl of the supernatant was transferred to a fresh glass vial for LC/MS analysis. The quality control (QC) sample was prepared by mixing an equal aliquot of the supernatants from all the samples.

UHPLC separation was carried out using a 1,290 Infinity series UHPLC System (Agilent Technologies), equipped with a UPLC BEH Amide column (2.1 × 100 mm, 1.7 μm, Waters). The mobile phase consisted of 25 mmol/l ammonium acetate and 25 mmol/l ammonia hydroxide in water (pH = 9.75) (A) and acetonitrile (B). The analysis was carried out with elution gradient as follows: 0–0.5 min, 95% B; 0.5–7.0 min, 95-65% B; 7.0–8.0 min, 65–40% B; 8.0–9.0 min, 40% B; 9.0–9.1 min, 40–95% B; and 9.1–12.0 min, 95% B. The column temperature was 25°C. The auto-sampler temperature was 4°C, and the injection volume was 1 μl (pos) or 1 μl (neg).

TripleTOF 6,600 mass spectrometry (AB Sciex) was used for its ability to acquire MS/MS spectra on an information-dependent acquisition (IDA) during in an LC/MS experiment. In this mode, the acquisition software (Analyst TF 1.7, AB Sciex) continuously evaluates the full scan survey MS data as it collects and triggers the acquisition of MS/MS spectra depending on preselected criteria. In each cycle, the most intensive 12 precursor ions with intensity above 100 were chosen for MS/MS at a collision energy (CE) of 30 eV. The cycle time was 0.56 s. ESI source conditions were set as following: gas 1 as 60 psi, gas 2 as 60 psi, curtain gas as 35 psi, source temperature as 600°C, declustering potential as 60 V, and ion spray voltage floating (ISVF) as 5000 V or -4000 V in positive or negative modes.

Data preprocessing and annotation: MS raw data (.wiff) files were converted to the mzXML format by ProteoWizard and processed by R package XCMS (version 3.2) (Smith et al., 2006). The process includes peak deconvolution, alignment, and integration. Minfrac and cut off are set as 0.5 and 0.6, respectively. An in-house MS2 database was applied for metabolite identification (Kuhl et al., 2012).

## 3 Results

### 3.1 The evaluation of palmatine, berberine, baicalin, and geniposide bound to MD-2

**FIGURE 1:**
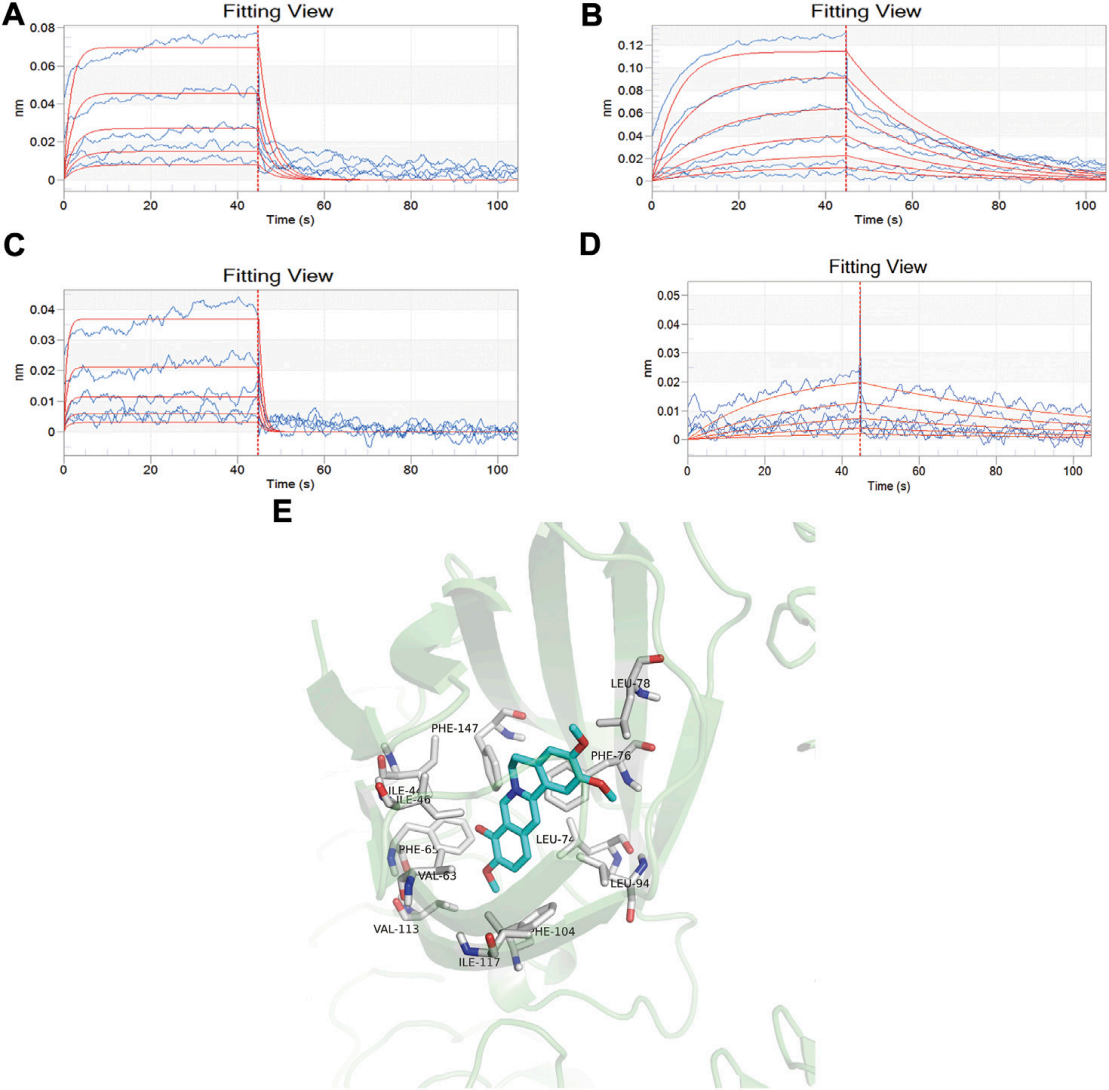
Screening of high-affinity active components by bio-layer interferometry assay and molecule docking. **(A)** Analysis of baicalin interaction with MD-2 by bio-layer interferometry assay, and the dissociation equilibrium constant in response to the concentration of baicalin varied from 0.0313, 0.0625, 0.125, 0.25, and 0.5 mM/ml; **(B)** Analysis of palmatine interaction with MD-2 by bio-layer interferometry assay, and the dissociation equilibrium constant in response to the concentration of palmatine varied from 0.0156, 0.0313, 0.0625, 0.125, 0.25, and 0.5 mM/ml; **(C)** Analysis of berberine interaction with MD-2 by bio-layer interferometry assay, and the dissociation equilibrium constant in response to the concentration of berberine varied from 0.0313, 0.0625, 0.125, 0.25, and 0.5 mM/ml; **(D)** Analysis of geniposide interaction with MD-2 by bio-layer interferometry assay, and the dissociation equilibrium constant in response to the concentration of berberine varied from 0.0625, 0.125, 0.25, 0.5, and 1.0 mM/ml. **(E)** Docking mode of MD2 protein and palmatine.

The affinity constants of baicalin, palmatine, berberine, and geniposide that physically interacted with MD-2 were determined by global fitting analysis of a 1:1 binding interaction model. The KD values of baicalin, palmatine, berberine, and geniposide with MD-2 were 5.52E-04, 1.63E-04, 6.42E-04, and 4.83E-04, respectively, which demonstrated a good binding capacity of baicalin, palmatine, berberine, and geniposide to MD-2 (Figures 1A–D). Gardenoside and berberine showed a rapid bonding and dissociation mode with MD-2. The order of binding affinity with MD-2 was palmatine, geniposide, baicalin, and berberine.

Molecular docking was used to further verify the affinity of MD-2 and the effective components of baicalin, palmatine, berberine, and geniposide. The result indicated that the four effective components are all high-affinity components to MD-2 (see Table 2). Figure 1E showed the example of palmatine to target protein MD-2. Baicalin binds to MD-2 by hydrogen bonds, and baicalin and berberine bind to MD-2 by π–π bond, and the four compounds bind to MD-2 by hydrophobic bonds.

**TABLE 2:** Docking of the effective components to MD-2 (PDB code: 3vq2).

Compound	Structure	Docking interaction
Baicalin	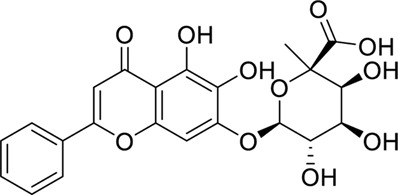	π–π stacking: 3.8 and 5.1 Å, yellow dotted line, PHE65 and PHE104
		H-Bond: 2.5 Å, TYR102, purple dotted line
		Hydrophobicity: VAL63, LEU71, LEU74, PHE76, VAL113, THR115, ILE117, LEU146, and PHE147
Berberine	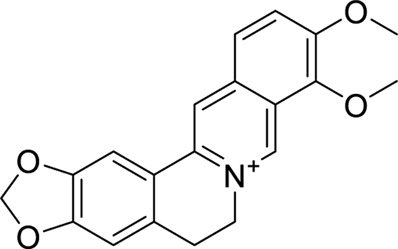	π–π stacking:3.9 and 5.5 Å, yellow dotted line, PHE76 and 104
		H-Bond: NA
		Hydrophobicity: ILE44, PHE65, THR115, ILE117, and PHE147
Geniposide	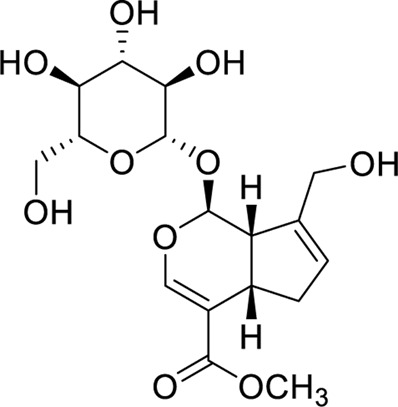	π–π stacking: NA
		H-Bond: 2.9 Å, TYR102
		Hydrophobicity: ILE44, ILE46, VAL61, VAL63, PHE65, LEU71, PHE76, PHE104, VAL113, and PHE147
Palmatine	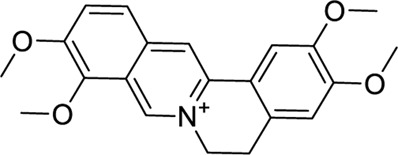	π–π stacking: NA
		H-Bond: NA
		Hydrophobicity: ILE44, ILE46, VAL63, PHE65, LEU74, PHE76, LEU78, LEU94, PHE104, VAL113, ILE117, and PHE147

### 3.2 The pathway analysis of palmatine, berberine, baicalin, and geniposide against sepsis

**FIGURE 2:**
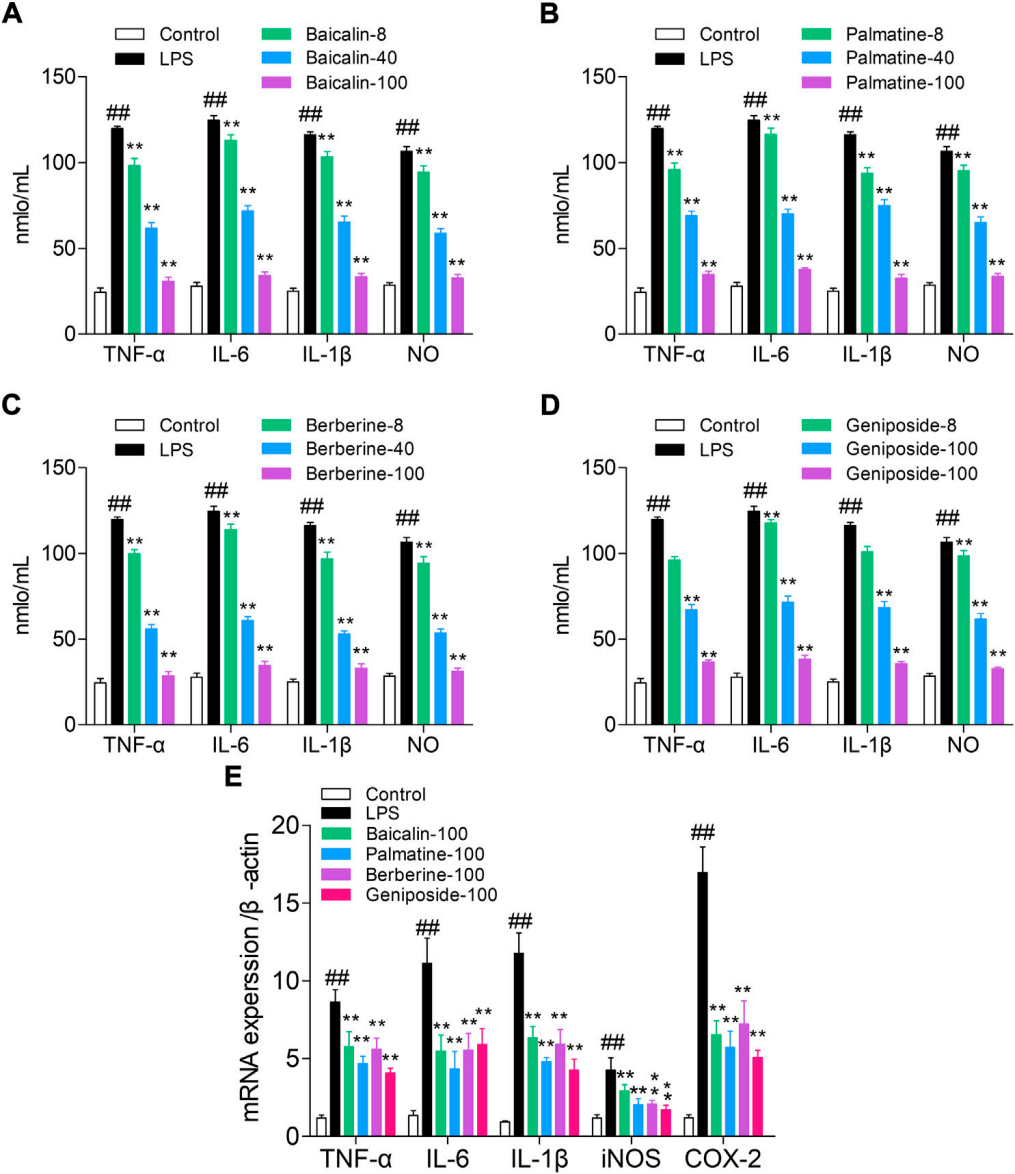
Study on the pathway of active components with high affinity, and the quantitative units in the figures (for example, 8, 40, and 100) were μg/ml. **(A)** Effect of baicalin on cytokines (NO, TNF-α, IL-6, and IL-1β) in RAW 264.7 cells; **(B)** Effect of palmatine on cytokines (NO, TNF-α, IL-6, and IL-1β) in RAW 264.7 cells; **(C)** Effect of berberine on cytokines (NO, TNF-α, IL-6, and IL-1β) in RAW 264.7 cells; **(D)** Effect of geniposide on cytokines (NO, TNF-α, IL-6, and IL-1β) in RAW 264.7 cells; **(E)** mRNA expression of iNOS, IL-1β, IL-6, TNF-α, and COX-2 in RAW 264.7 cells treated with baicalin, palmatine, berberine, or geniposide.

The ELISA method was used to detect the levels of NO, TNF-α, IL-6, and IL-1β. Compared with those of the control group, the levels of NO, IL-1β, IL-6, and TNF-α were significantly increased in the LPS group (all *p* < 0.01). Cells treated with LPS and baicalin, palmatine, berberine, or geniposide showed that they can significantly decrease NO, IL-1β, IL-6, and TNF-α levels in the LPS group (*p* < 0.01), while 8 μg/ml palmatine could decrease the IL-6 level in the LPS group (*p* < 0.05), and 8 μg/ml geniposide had no effect on NO and IL-6 levels in the LPS group (*p* > 0.05). (Figures 2A–D).

RT-q PCR was used to analyze the IL-1β, TNF-α, iNOS, IL-6, and COX-2 mRNA expression levels. Compared with the control group, the LPS group showed high levels of iNOS, IL-1β, IL-6, TNF-α, and COX-2 mRNA expression (all *p* < 0.01), and while treated with LPS and baicalin, palmatine, berberine, or geniposide, the iNOS, IL-1β, IL-6, TNF-α, and COX-2 mRNA expression levels were significantly decreased in the LPS group (all *p* < 0.01). (Figure 2E).

**FIGURE 3:**
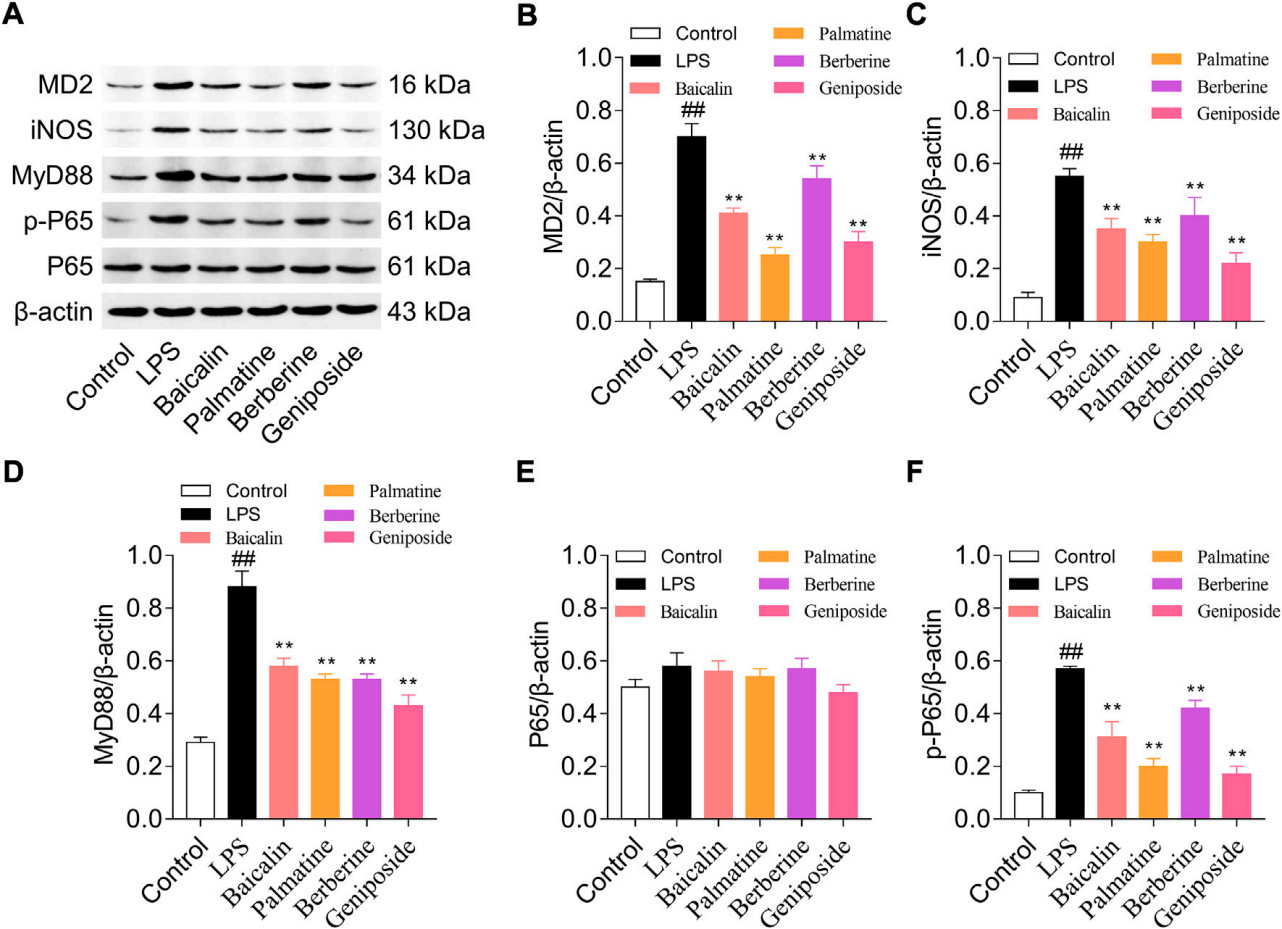
MD-2, MYD88, p65, p-p65, and iNOS protein expression levels in RAW 264.7 cells treated with baicalin, palmatine, berberine, or geniposide. **(A)** Western blot electrophoresis results, **(B)** relative protein expression of MD-2, **(C)** relative protein expression of iNOS, **(D)** relative protein expression of MYD88, **(E)** relative protein expression of p65, and **(F)** relative protein expression of p-p65.

Western blotting was used to analyze the protein expression levels of MD-2, MyD88, NF-κB p65, NF-κB p-p65, and iNOS. The result indicated that MD-2, MyD88, p-p65, and iNOS expression levels were upregulated in the LPS group than in the control group (all *p* < 0.01), and while treated with LPS and baicalin, palmatine, berberine, or geniposide, MD-2, MyD88, p-p65, and iNOS expression levels were downregulated in the LPS group (all *p* < 0.01) (Figure 3). (The unedited blot for Figure 3 see Supplementary Figure S2)

Most importantly, the results showed that the four components have anti-sepsis effect through the LPS-TLR4/MD-2-NF-κB signaling pathway. The four components were prepared as active component formulation (ACF) according to the original content of HJD prescription (Chen G. et al., 2016; Jing et al., 2016). Then, the anti-sepsis effect and mechanism of ACF were explored and compared with those of HJD.

### 3.3 Pharmacodynamics and metabolomics analysis of ACF and HJD

#### 3.3.1 Histopathological analysis of the heart, lung, liver, kidney, and colon tissues

**FIGURE 4:**
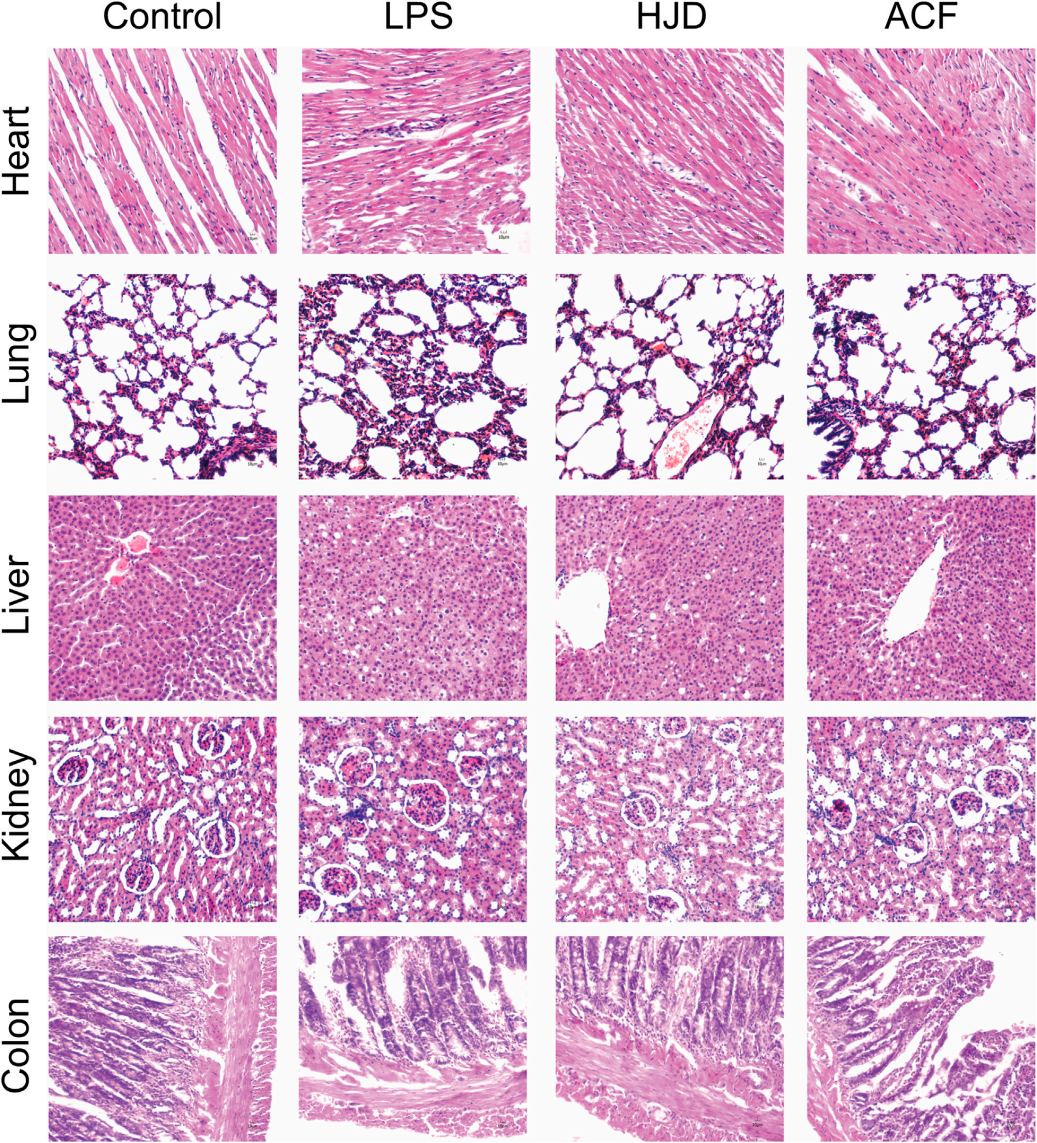
Pathological changes of the heart, liver, lung, kidney, and colon tissues of rats in each group by histological analysis (X200). (Pathological results: compared to the normal group, the heart, lung, liver, kidney, and colon tissues all exhibited a large number of inflammatory cell infiltration in the LPS group. Therefore, for heart tissues, the myocardial cells in the LPS group were disordered in arrangement, and the transverse striations were blurry and broken. The lung injury in the LPS group was alveolar congestion and edema; the liver cells had an irregular and disordered cord-like arrangement and unclear boundaries in LPS group; the glomeruli showed abnormal size and swelling with unclear boundaries in the LPS group; the colonic mucosa disruption, goblet cell loss, and epithelial shedding were observed in the LPS group, and ACF and HJD groups had significant improvement effects on them.).

Histopathological examination was performed for multiple organs of rats in each group. Compared to the normal group, the heart, lung, liver, kidney, and colon tissues exhibited a large number of inflammatory cell infiltration in the LPS group. Therefore, for heart tissues, the myocardial cells in the LPS group were disordered in arrangement, and the transverse striations were blurry and broken. The lung injury in the LPS group was characterized by alveolar congestion and edema; the liver cells had an irregular and disordered cord-like arrangement and unclear boundaries in the LPS group; the glomeruli showed abnormal size and swelling with unclear boundaries in the LPS group; and colonic mucosa disruption, goblet cell loss, and epithelial shedding were observed in the LPS group. These phenomena were reversed in the HJD and ACF groups, indicating that ACF and HJD can play a protective role against organ damage caused by sepsis. (Figure 4).

#### 3.3.2 ELISA assay for the NO, IL-1β, IL-6, and TNF-α levels

**FIGURE 5:**
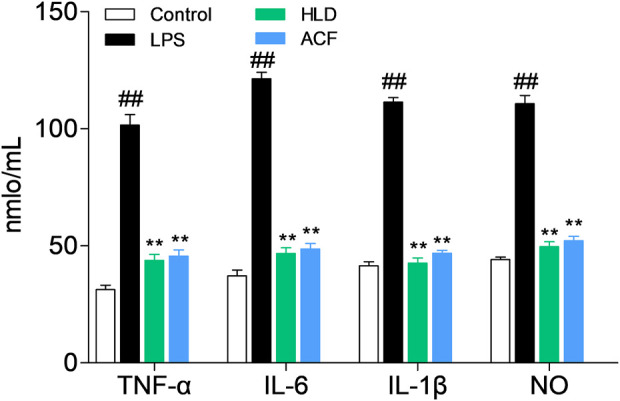
Effect of ACF on cytokines (NO, IL-1β, IL-6, and TNF-α) in rats.

The ELISA method was used to detect the NO, IL-1β, IL-6, and TNF-α levels of serum from rats in each group. Compared with those in the control group, the levels of NO, IL-1β, IL-6, and TNF-α were significantly increased in the LPS group (all *p* < 0.01). Rats treated with HJD or ACF showed that they can significantly decrease NO, IL-1β, IL-6, and TNF-α levels in the LPS group (*p* < 0.01). Both HJD and ACF showed good anti-sepsis activity *in vivo*. (Figure 5).

#### 3.3.3 Metabolomics

**FIGURE 6:**
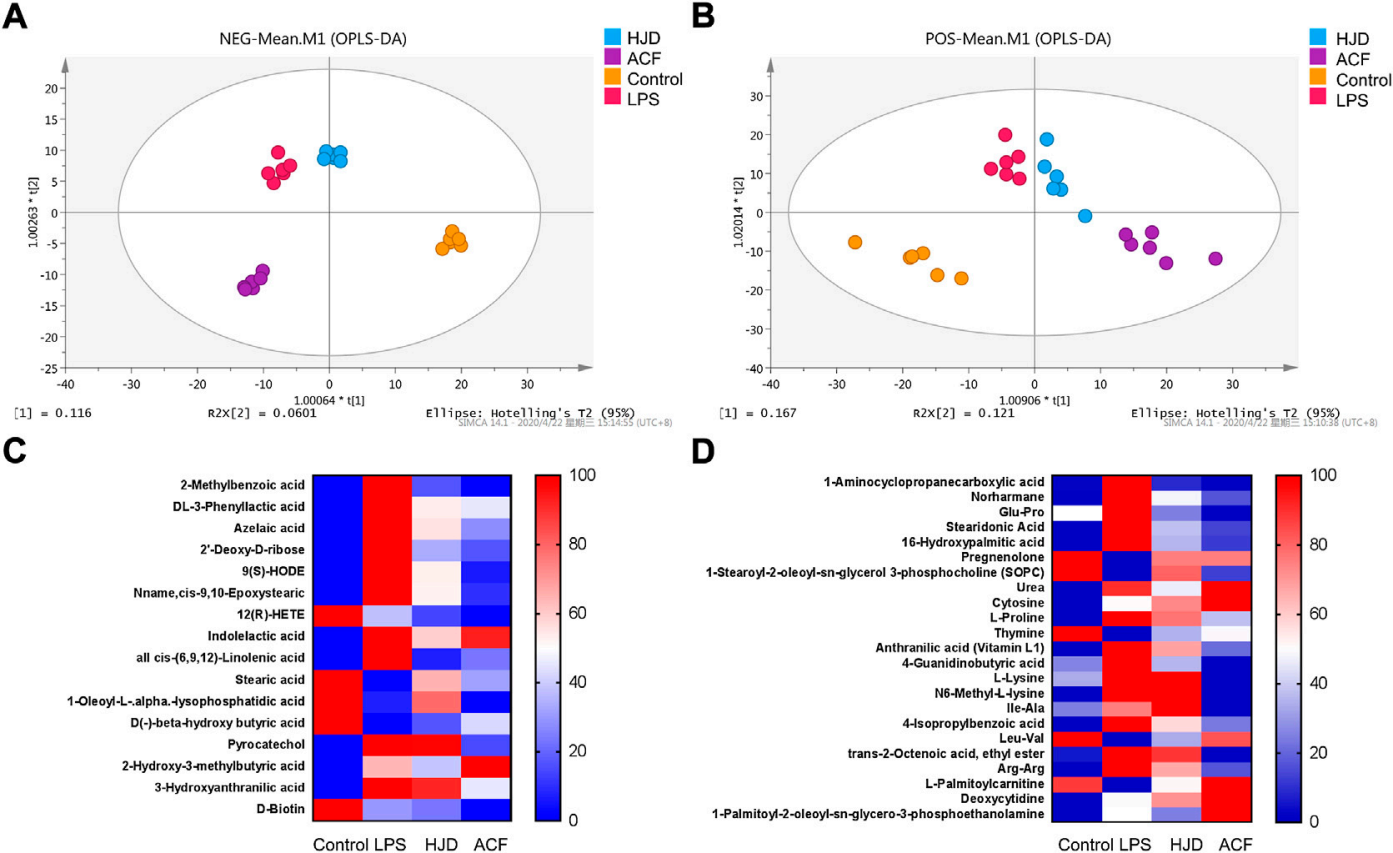
Score plots according to OPLS-DA analysis based on MS data from the serum of all groups and heatmap visualization of the differential metabolites. **(A)** negative ion mode; **(B)** positive ion mode. **(C)** negative ion mode; and **(D)** positive ion mode. Columns represent groups, and rows represent metabolites, and the color indicates metabolite quantities, where red means upregulated and blue means downregulated.

In this study, 1,160/1,228 peaks were detected, and 309/249 metabolites were left for positive/negative ion mode after relative standard deviation denoising. Then, the missing values were filled up by half of the minimum value. Also, the internal standard normalization method was used in this data analysis. The final dataset containing the information of peak number, sample name, and normalized peak area was imported to the SIMCA 14.1 software package (Sartorius Stedim Data Analytics AB, Umea, Sweden) for multivariate analysis. Data were scaled and logarithmically transformed to minimize the impact of both noise and high variance of the variables (Supplementary Tables S1, S2). After these transformations, supervised orthogonal projections to latent structures-discriminate analysis (OPLS-DA) was applied in order to visualize group separation and find significantly changed metabolites, and the score plots for negative and positive ion modes both presented a clear clustering of the control, LPS, HJD, and ACF groups (Figures 6A,B) with a good goodness of fit (R2X (cum) = 0.494, R2Y (cum) = 0.893, Q2 (cum) = 0.639; R2X (cum) = 0.679, R2Y (cum) = 0.988, Q2 (cum) = 0.467), indicating that the models were good. Thus, the value of variable importance in the projection (VIP) of the first principal component in OPLS-DA analysis was obtained. It summarizes the contribution of each variable to the model. The metabolites with VIP>1 and *p*< 0.05 (one-way ANOVA test) were considered significantly changed metabolites. For reliability of the conclusion, only metabolites verified by the MS2 database were selected for further study, and the heatmaps of these metabolites from negative/positive ion modes were generated, demonstrating the relative increase (red) or decrease (blue) (Figures 6C,D).

Significantly increased 2-methylbenzoic acid, DL-3-phenyllactic acid, azelaic acid, 2′-deoxy-D-ribose, 9(S)-HODE, Nname, cis-9,10-epoxystearic acid, indolelactic acid, all cis-(6,9,12)-linolenic acid, pyrocatechol, 2-hydroxy-3-methylbutyric acid, 3-hydroxyanthranilic acid, 1-aminocyclopropanecarboxylic acid, norharmane, glu-Pro, stearidonic acid, 16-hydroxypalmitic acid, urea, cytosine, L-proline, anthranilic acid (vitamin L1), 4-guanidinobutyric acid, L-lysine, N6-methyl-L-lysine, Ile-Ala, 4-isopropylbenzoic acid, trans-2-octenoic acid, ethyl ester, Arg–Arg, 1-palmitoyl-2-oleoyl-sn-glycero-3-phosphoethanolamine, and deoxycytidine and significantly decreased 12(R)-HETE, stearic acid, 1-oleoyl-L-.alpha.-lysophosphatidic acid, D(-)-beta-hydroxy butyric acid, D-Biotin, pregnenolone, 1-stearoyl-2-oleoyl-sn-glycerol 3-phosphocholine (SOPC), thymine, Leu-Val, and L-palmitoylcarnitine were observed in the LPS group compared with the control group, while these effects induced by LPS could be reversed by HJD or ACF except 2-hydroxy-3-methylbutyric acid, D-biotin, cytosine, deoxycytidine, and 1-palmitoyl-2-oleoyl-sn-glycero-3-phosphoethanolamine (Table 3). To our interest, some metabolites, such as 3-hydroxyanthranilic acid, can be reversed by ACF after LPS induction, while HJD cannot; these metabolites would be further discussed (see discussion).

**TABLE 3:** Mass databases of 39 potential differential metabolites.

No.	Ion mode	R.T.(s)	m/z	Metabolite	Control group	LPS group	HJD group	ACF group
1	ESI(-)	100.315	135.0432	2-methylbenzoic acid	0.33 ± 0.03	0.45 ± 0.06^##^	0.35 ± 0.04**	0.33 ± 0.06**
2	ESI(-)	113.455	165.0535	DL-3-phenyllactic acid	0.24 ± 0.04	3.37 ± 0.92^##^	1.91 ± 0.56**	1.66 ± 0.24**
3	ESI(-)	318.5205	187.0951	Azelaic acid	0.11 ± 0.03	0.29 ± 0.07^##^	0.21 ± 0.05*	0.16 ± 0.04**
4	ESI(-)	145.6005	267.1046	2′-deoxy-D-ribose	0.01 ± 0	0.07 ± 0.02^##^	0.03 ± 0.01**	0.02 ± 0.01**
5	ESI(-)	64.448	295.2234	9(S)-HODE	0.52 ± 0.13	2.13 ± 0.55^##^	1.37 ± 0.28**	0.62 ± 0.14**
6	ESI(-)	46.35	297.2397	Nname, cis-9,10-epoxystearic acid	3.01 ± 0.62	12.22 ± 2.82^##^	7.81 ± 2.06**	3.96 ± 0.59**
7	ESI(-)	46.265	319.2247	12(R)-HETE	69.74 ± 17.74	27.06 ± 7.07^##^	11.03 ± 0.61*	1.35 ± 0.4**
8	ESI(-)	147.447	204.0637	Indolelactic acid	0.1 ± 0.02	0.52 ± 0.1^##^	0.35 ± 0.09**	0.49 ± 0.13
9	ESI(-)	43.0445	277.2139	all cis-(6,9,12)-linolenic acid	24.67 ± 4.35	36.52 ± 9.59^#^	25.51 ± 7.11*	27.42 ± 3.6
10	ESI(-)	43.038	283.2607	Stearic acid	161.05 ± 16.63	122.55 ± 11.03^##^	147.5 ± 10.49*	134.71 ± 17.46
11	ESI(-)	231.0535	457.2287	1-Oleoyl-L-.alpha.-lysophosphatidic acid	0.22 ± 0.05	0.09 ± 0.03^##^	0.19 ± 0.05**	0.08 ± 0.02
12	ESI(-)	218.062	103.0391	D(-)-beta-hydroxy butyric acid	6.23 ± 1.21	0.52 ± 0.12^##^	1.50 ± 0.41	2.94 ± 0.75**
13	ESI(-)	22.936	109.0301	Pyrocatechol	0.1 ± 0.02	2.27 ± 0.62^##^	2.23 ± 0.65	0.42 ± 0.12**
14	ESI(-)	152.815	117.0541	2-hydroxy-3-methylbutyric acid	0.16 ± 0.04	0.39 ± 0.08^##^	0.30 ± 0.06	0.52 ± 0.1*
15	ESI(-)	22.427	189.9920	3-hydroxyanthranilic acid	0.09 ± 0.02	1.01 ± 0.17^##^	0.93 ± 0.26	0.51 ± 0.12**
16	ESI(-)	152.815	281.0319	D-biotin	0.4 ± 0.05	0.19 ± 0.05^##^	0.17 ± 0.05	0.1 ± 0.02**
17	ESI(+)	314.117	143.0823	1-aminocyclopropanecarboxylic acid	0.85 ± 0.28	4.06 ± 2.08^##^	1.08 ± 0.59**	0.79 ± 0.37**
18	ESI(+)	44.289	169.0766	Norharmane	0.12 ± 0.05	0.6 ± 0.14^##^	0.35 ± 0.25*	0.2 ± 0.15**
19	ESI(+)	311.5205	245.1079	Glu-Pro	0.08 ± 0.02	0.1 ± 0.02^#^	0.07 ± 0.01**	0.06 ± 0.01**
20	ESI(+)	46.6015	277.218	Stearidonic acid	0.07 ± 0.01	1.15 ± 0.67^##^	0.48 ± 0.43*	0.22 ± 0.24**
21	ESI(+)	46.964	295.2281	16-hydroxypalmitic acid	0.1 ± 0.06	1.39 ± 0.88^##^	0.56 ± 0.52*	0.26 ± 0.29**
22	ESI(+)	42.168	299.2385	Pregnenolone	0.1 ± 0.01	0.06 ± 0.01^##^	0.09 ± 0.03*	0.09 ± 0.02*
23	ESI(+)	125.8675	810.6072	1-stearoyl-2-oleoyl-sn-glycerol 3-phosphocholine (SOPC)	16.41 ± 2.92	9.53 ± 2.2^##^	15.05 ± 4.25**	10.43 ± 1.08
24	ESI(+)	102.226	61.04035	Urea	0.72 ± 0.15	1.73 ± 0.35^##^	1.24 ± 0.17*	1.84 ± 0.48
25	ESI(+)	193.0075	112.0513	Cytosine	4.72 ± 0.81	7.23 ± 1.74^#^	8.37 ± 1.75	9.71 ± 0.64*
26	ESI(+)	290.792	116.0717	L-proline	6.02 ± 0.82	9.49 ± 1.01^##^	8.69 ± 1.1	7.32 ± 0.88**
27	ESI(+)	98.944	127.0506	Thymine	0.9 ± 0.15	0.47 ± 0.03^##^	0.62 ± 0.15	0.68 ± 0.17*
28	ESI(+)	205.108	138.0553	Anthranilic acid (vitamin L1)	0.2 ± 0.06	0.76 ± 0.21^##^	0.58 ± 0.36	0.32 ± 0.12**
29	ESI(+)	336.4505	146.0925	4-guanidinobutyric acid	0.7 ± 0.28	1.24 ± 0.47^#^	0.77 ± 0.38	0.51 ± 0.12**
30	ESI(+)	254.044	146.1072	L-lysine	0.07 ± 0.01	0.09 ± 0.02^#^	0.09 ± 0.01	0.06 ± 0.01**
31	ESI(+)	254.3765	202.1557	N6-methyl-L-lysine	0.04 ± 0.01	0.05 ± 0^##^	0.05 ± 0.01	0.04 ± 0.01**
32	ESI(+)	217.626	203.1397	Ile-Ala	0.05 ± 0.01	0.07 ± 0.02^#^	0.08 ± 0.02	0.04 ± 0.01**
33	ESI(+)	91.33	225.113	4-iopropylbenzoic acid	0.08 ± 0.01	0.29 ± 0.11^##^	0.2 ± 0.08	0.13 ± 0.02**
34	ESI(+)	355.503	231.1714	Leu-Val	0.09 ± 0.03	0.03 ± 0.01^##^	0.05 ± 0.01	0.08 ± 0.04*
35	ESI(+)	125.889	234.1501	trans-2-octenoic acid, ethyl ester	0.15 ± 0.08	0.48 ± 0.12^##^	0.44 ± 0.24	0.13 ± 0.07**
36	ESI(+)	272.7375	372.2398	Arg-Arg	0.02 ± 0.01	0.08 ± 0.05^##^	0.06 ± 0.03	0.03 ± 0.01**
37	ESI(+)	155.052	400.3446	L-palmitoylcarnitine	1.41 ± 0.4	0.44 ± 0.21^##^	1.01 ± 0.67	1.55 ± 0.49**
38	ESI(+)	193.006	455.1922	Deoxycytidine	16.55 ± 3.64	26.86 ± 6.9^#^	31.48 ± 8.39	37.55 ± 3.72*
39	ESI(+)	131.486	718.5384	1-palmitoyl-2-oleoyl-sn-glycero-3-phosphoethanolamine	0.03 ± 0.01	0.05 ± 0.01^##^	0.04 ± 0.01	0.07 ± 0.02**

Compared to the control group, ^#^ *p* < 0.05, ^##^ *p* < 0.01; compared to the LPS group, *p < 0.05 and **p < 0.01.

**FIGURE 7:**
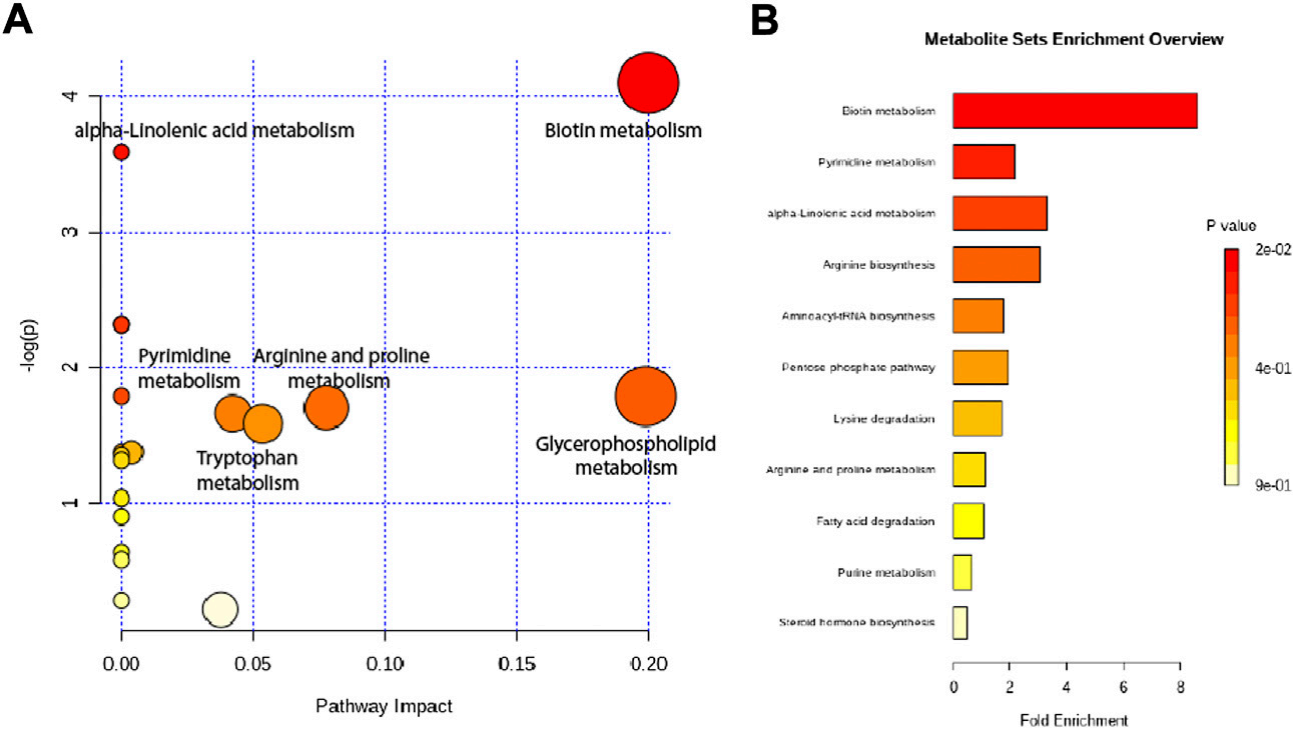
Metabolic pathway analysis of altered metabolites. **(A)** Pathways visualized as bubble plots. **(B)** Pathways visualized as enrichment overview.

Altered metabolites were subjected to pathway analysis using MetaboAnalyst (http://www.metaboanalyst.ca) and KEGG (http://www.genome.jp/kegg/) (Figures 7A,B). These metabolites were distributed in the related pathways of biotin metabolism, alpha-Linolenic acid metabolism, glycerophospholipid metabolism, arginine and proline metabolism, pyrimidine metabolism, and tryptophan metabolism.

## 4 Discussion

Homologous dimerization is the structural basis for recruitment of protein to initiate downstream inflammatory pathways, and the binding of LPS to TLR4/MD-2 complex would result in dimerization of the TLR4 receptor, where the lipid chains and phosphate groups of Lipid A contribute directly to dimerization. Furthermore, CD14 and LBP could promote the recognition of LPS by TLR4/MD-2 heterodimer (Miyake, 2006). MD-2 played a key role in the recognition of LPS, and TLR4 had functions in triggering inflammatory signal transduction (Park JE. et al., 2009; Park BS. et al., 2009). LPS-MD-2/TLR4 dimerization was formed to induce the aggregation of MyD88 and TLR4 through stimulating TLR4/MD-2 by LPS, followed by the recruitment of IL-1R-associated kinase-4 (IRAK-4), IRAK-1, and IRAK-2; and the phosphorylation of IRAK-1 and IRAK-2 were induced by IRAK-4 to form new protein complexes with tumor necrosis factor-α receptor-associated factor-6 (TRAF-6) after dissociating from the MyD88-TLR4 complex, and the transforming growth factor-β-activated kinase 1 (TAK1) was activated and eventually the activation of signaling cascades that promote the activation of mitogen-associated protein kinases (MAPKs), NF-κB, and subsequent expression of pro-inflammatory mediators (Keating et al., 2007). Overall, the LPS-TLR4/MD-2-NF-κB pathway is recognized as a key pathway of sepsis; thus, the anti-sepsis study of the active components from HJD was conducted based on the MD-2 target.

SPR is a biosensing analysis technology for analyzing the interaction between biomolecules, which could detect the specificity, binding intensity, binding process, speed of binding/dissociation, binding sites, and binding sequence of biomolecules in real- time (Radhakrishnan and Poltronieri, 2017). As an important auxiliary method in the field of new drug research, molecular docking played an extremely significant role in the study of the mechanism and development of new drugs (Pagadala et al., 2017). This study found that the four active components can bind to MD-2 with the aid of the SPR and molecular docking technique, suggesting that baicalin, palmatine, berberine, or geniposide showed strong affinity binding with MD-2. The results of SPR showed that palmatine exhibited the strongest affinity with MD-2. However, the molecular docking results indicated that palmatine only bonded with MD-2 by a hydrophobic bond. It suggests that the hydrophobic binding of the compound to MD-2 is the key to antagonize the effect of MD-2.

In the early stage of sepsis, a large amount of pro-inflammatory factors was released, resulting in systemic inflammatory reaction. IL-6 was a cytokine secreted by the body in the early stage of sepsis, which played an important role in development and prognosis of septic patients (Oda et al., 2005). The anti-IL-6 monoclonal antibody was demonstrated to have a protective effect on CLP sepsis rats and improve the survival rate of sepsis rats significantly (Riedemann et al., 2003). Therefore, it was also confirmed that anti-IL-6 receptor monoclonal antibodies can ameliorate the inflammation in burn mice in addition to anti-IL-6 monoclonal antibodies (Pallua et al., 2003) As the studies of IL-6 signal transduction pathways continue, IL-6 had emerged as a potential target for treatment of sepsis. TNF-α, as an important mediator in the pathogenesis of sepsis and septic shock, could be resulting in turning the uncontrolled inflammatory cascade reaction and eventually leads to endotoxin shock, tissue damage, and multiple organ dysfunction, which also became an important research mediator for sepsis treatment (Herzum and Renz, 2008). NO could normally prevent vascular spasm and thrombosis, but the high concentration of NO would cause body damage through lipid peroxidation induced by iNOS under the condition of sepsis (Chen and Wei, 2021; Shen et al., 2021). NO could cause microcirculation disorder and vascular permeability decrease in sepsis that play an important role in the early onset of sepsis. It had been shown that cell *pyroptosis* is closely related to inflammatory response waterfall and organ damage caused by sepsis. Also, excessive activation induced by cell *pyroptosis* causes the release of a large amount of IL-1β, which would lead to cause inflammatory storms and organ damage once again (Ismi et al., 2017; Cavaillon, 2018). COX-2 is an inducible enzyme with enhanced the expression in tissue injury and inflammation. The TLR4 pathway plays an important role for mediating inflammation induced by LPS, and the inflammatory effect can be exerted by activating and regulating downstream COX-2. NF-κB is the key intermediate link for TLR4 involved in the regulation of COX-2. The *in vitro* studies found that baicalin, palmatine, berberine, or geniposide could inhibit the release of NO, IL-1β, IL-6, and TNF-α and reduce the mRNA expression of iNOS, IL-1β, IL-6, TNF-α, and COX-2, respectively. The four compounds could markedly decrease the protein expression of MD-2, MyD88, NF-κB p65, NF-κB p-p65, and iNOS in the LPS-TLR4/MD-2-NF-κB pathway. The results indicated that the compounds played anti-sepsis effect through inhibiting the release of NO, IL-1β, IL-6, and TNF-α mediated by LPS and regulating the LPS-TLR4/MD-2-NF-κB pathway by hydrophobic binding to the key protein MD-2.

A previous study by this group had demonstrated the therapeutic effects of HJD on sepsis, but HJD had hundreds of components, and how to effectively control its true anti-sepsis active components was a difficult point, and screening the active anti-sepsis components from HJD to form the active component formulation might be an effective way to expand the application of traditional Chinese medicine. Also, the *in vitro* studies indicated that baicalin, palmatine, berberine, and geniposide can all play an anti-sepsis role by mediating the LPS-TLR4/MD-2-NF-κB pathway, where the effect was consistent. Based on the *in vitro* research, the active components (berberine, palmatine, baicalin, and geniposide) from HJD were re-formed into a new formulation in order to ensure anti-sepsis efficacy and achieve quality standardization at the same time. Thus, the *in vivo* experiments for sepsis treatment by ACF and HJD were studied simultaneously.

Indeed, there was a close relationship between organ injury and development of sepsis. The liver played an immense role in catabolism, metabolism, synthesis, secretion, detoxification, immunity, and defense. Acute lung injury was a common complication of sepsis. Accordingly, the lung and liver were the most vulnerable organs to the inflammatory cascade triggered by sepsis. Sepsis could cause a series of inflammatory responses that induce gastrointestinal dysfunction, such as abdominal distension, constipation, vomiting, abdominal pain, and diarrhea. 64% of sepsis patients may be associated with myocardial injury, and its severity was closely related to the early mortality of sepsis (Pulido et al., 2012; Frencken et al., 2018). A study also had found that 47.5% of patients with sepsis develop acute kidney injury (Bagshaw et al., 2007). The study found that both the ACF and HJD can inhibit the release of inflammatory factors (NO, IL-1β, IL-6, and TNF-α), reduce inflammatory cell infiltration, and induce organ damage by sepsis.

The body would go through a state of high-energy metabolism under sepsis. As the main energy supply substance, the abnormal changes of carbohydrate metabolites in the body could directly indicate the abnormal energy metabolism, while amino acids and lipids, as energy storage units, would indirectly reveal abnormal energy metabolism and also reflect the compensatory mechanism of maintaining energy metabolism homeostasis (Ryan and Robards, 2006). Our study found that ACF and HJD played an anti-sepsis effect mainly through regulation of amino acid metabolism (L-lysine, L-proline, 4-guanidinobutyric acid, N6-methyl-L-lysine, Ile-Ala, Leu-Val, Arg-Arg, 3-hydroxyanthranilic acid, 1-aminocyclopropanecarboxylic acid, Glu-Pro, and 4-isopropylbenzoic acid) and lipid metabolism (indolelactic acid, 2-methylbenzoic acid, DL-3-phenyllactic acid, trans-2-octenoic acid, ethyl ester, L-palmitoylcarnitine, 1-stearoyl-2-oleoyl-sn-glycerol 3-phosphocholine (SOPC), 1-palmitoyl-2-oleoyl-sn-glycero-3-phosphoethanolamine, azelaic acid, 9(S)-HODE, Nname, cis-9,10-epoxystearic acid, 12(R)-HETE, all cis-(6,9,12)-linolenic acid, stearic acid, stearidonic acid, and 16-hydroxypalmitic acid). Of course, most of the molecular mechanism for sepsis treatment by ACF and HJD were consistent, and what interests us the most was the differences between ACF and HJD.

3-hydroxyanthranilic acid exerted a cytotoxic effect, which inhibits mitochondrial respiration, promotes oxidative damage on proteins, and induces apoptosis (Mangas et al., 2016; Mangas et al., 2018). Anthranilic acid (vitamin L1) was a potentially toxic compound, known as one of uremic toxins, which could lead to a few diseases such as renal damage, cardiovascular disease, and chronic kidney disease (Duranton et al., 2012). It could also improve mood and cause changes in mental status, including reduced awareness, seizures, agitation, and confusion (Muszynska et al., 2015). Pyrocatechol had not been previously reported, and a study had shown that sepsis patients have high levels of catechol ammonia and endothelial injury markers, which were associated with severity of the disease and coagulation function (Hoppensteadt et al., 2015). L-Proline had been proposed to be a potential endogenous excitotoxin/neurotoxin, and studies on rats have shown that when injected into the brain, proline non-selectively destroyed pyramidal and granule cells (Nadler et al., 1988). In humans, thymine could regulate metabolic disorder through the beta-ureidopropionase deficiency pathway (Brown et al., 2016). 4-guanidinobutyric acid was a normal metabolite present in low concentrations, when lack of arginase and arginine accumulation in the body would lead to an increase of 4-guanidinobutyric acid (Marescau et al., 1995). N6-Methyl-L-lysine was known to be increased in patients with kidney injury or failure, while sepsis could also cause kidney damage (Lower et al., 1975). Neutrophil dysfunction was a key step in the pathophysiology of sepsis-associated organ dysfunction. Soroush et al. (2019) found that protein kinase C-δ (PKC-δ) tyrosine 155 mutations significantly reduce neutrophil migration to the lung in septic mice, where PKC-δ was a key regulator of neutrophil activation (Soroush et al., 2019). When sepsis occurs, PKC was activated, and L-palmitoylcarnitine could inhibit the activation of PKC at higher concentration (Nalecz et al., 2004). These metabolites abovementioned were regulated by ACF but not HJD. In contrast, indolelactic acid and urea were regulated by HJD but not ACF. Nevertheless, both of them had no obvious physiological effect with sepsis (Cala et al., 2018), and the variation in indolelactic acid and urea contents might be caused by the disturbance of amino acid metabolism. Moreover, some metabolites were elevated in the LPS, HJD, and ACF groups, which might be the compensatory mechanism of anti-sepsis by the body itself, such as deoxycytidine. A study had found that the serum deoxycytidine in patients with ulcerative colitis is higher than that in patients with Crohn’s disease (Kolho et al., 2017). However, those studies did not look at the effect of deoxycytidine on sepsis, and within humans, deoxycytidine participates in a number of enzymatic reactions. Thus, we speculated that an increase in deoxycytidine content may help treat sepsis.

Based on our findings, we formed a new formulation from HJD as protection against sepsis (Figure 7). The anti-sepsis pathway mechanism of ACF was consistent with that of HJD, while the molecular mechanism was slightly different between them, which also indicated that ACF might alleviate the damage to the body caused by HJD in a certain extent, and at the same time, it can also achieve the standardization of Chinese medicine formulation.

## 5 Conclusion

Overall, palmatine, berberine, baicalin, and geniposide have been identified as active components from HJD through their high affinity to MD-2, and their mechanisms in sepsis management were characterized by pharmacodynamics and metabolome analysis. This new formulation might be of great importance as anti-sepsis agents, providing new treatment strategies for sepsis management.

## Data Availability

The original contributions presented in the study are included in the article/Supplementary Material. Further inquiries can be directed to the corresponding authors.
