# Supplementary figures and images for: The interaction of MD-2 with small molecules in huanglian jiedu decoction play a critical role in the treatment of sepsis
Source: Front Pharmacol. 2022 Sep 9;13:947095. doi: 10.3389/fphar.2022.947095 (PMC9500189; doi:10.3389/fphar.2022.947095)

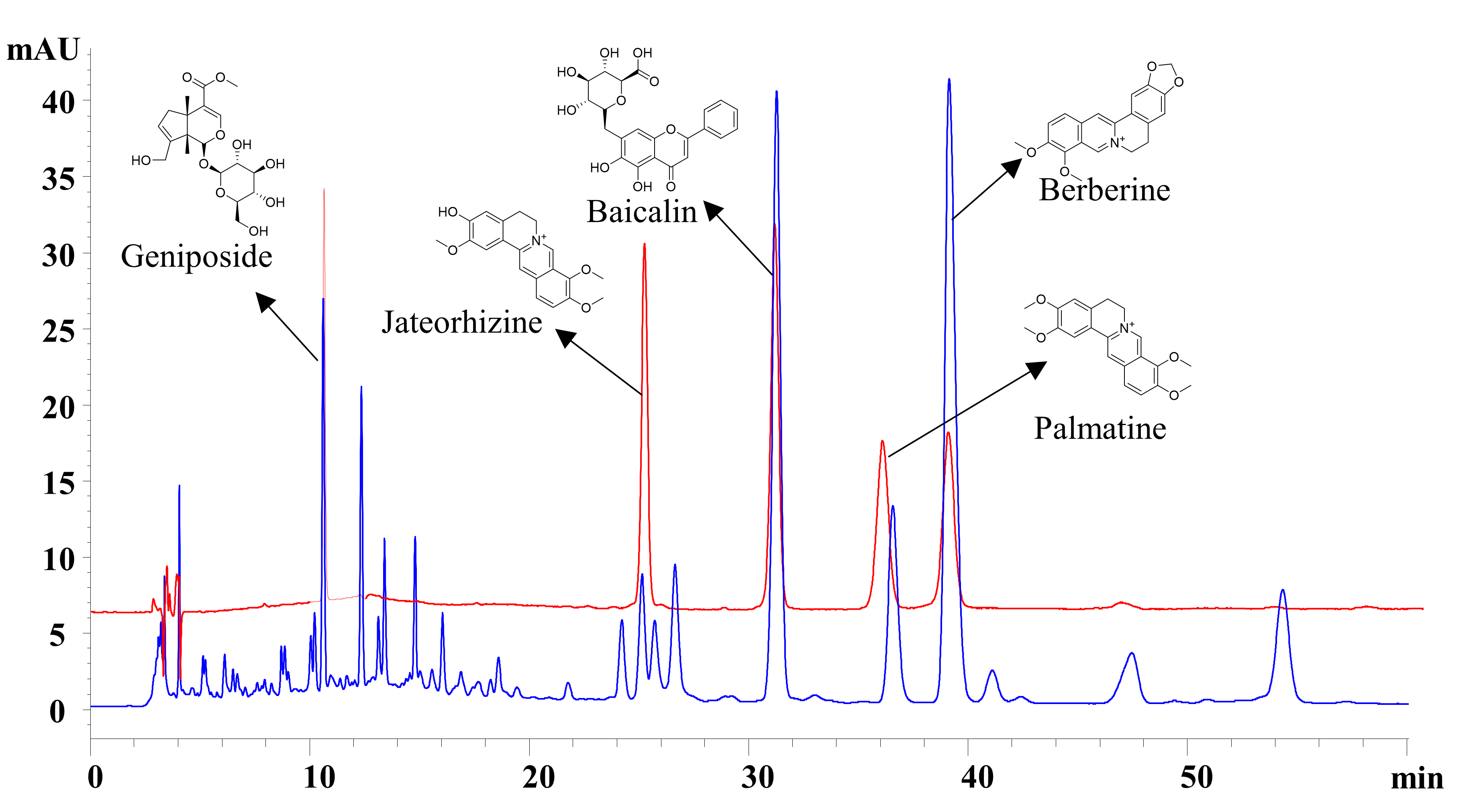

Supplement: Supplementary file 2 [file Image1.TIF]
