# Supplementary material for: The interaction of MD-2 with small molecules in huanglian jiedu decoction play a critical role in the treatment of sepsis
Source: Front Pharmacol. 2022 Sep 9;13:947095. doi: 10.3389/fphar.2022.947095 (PMC9500189; doi:10.3389/fphar.2022.947095)

Full unedited blot for **Figure S2**

(From top to bottom were described as β-actin, MD-2, MyD88, NF-κB p65, NF-κB p-p65, and iNOS, repeat 3 times; lane 1: Control, 2: LPS, 3: Baicalin-100, 4: Palmatine-100, 5: Berberine-100, 6: Geniposide-100

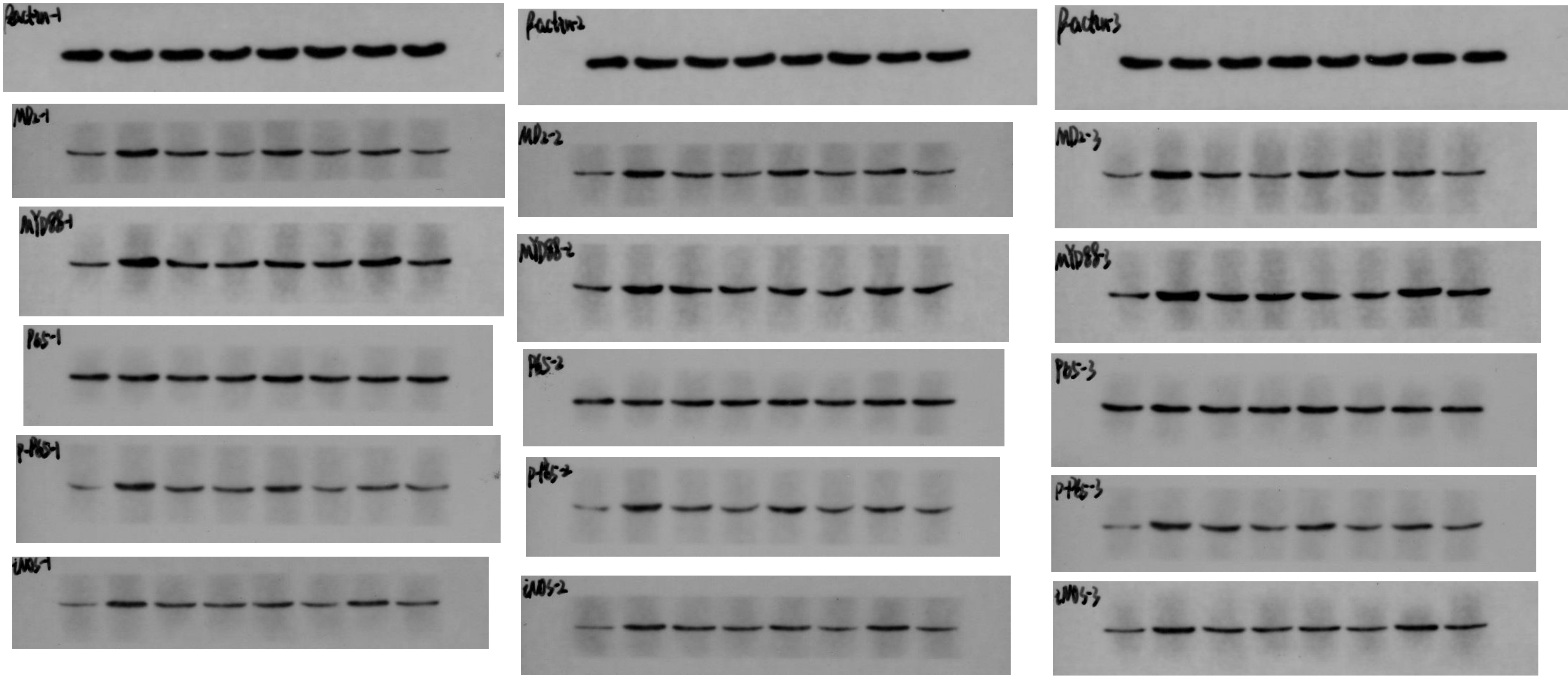

Supplement: Supplementary file 3 [file Image2.pdf]
